# Supplementary material for: Quantitative structural mechanobiology of platelet-driven blood clot contraction
Source: Nat Commun. 2017 Nov 2;8:1274. doi: 10.1038/s41467-017-00885-x (PMC5668372; doi:10.1038/s41467-017-00885-x)
Supplement: Supplementary file 1 — Supplementary Information [file 41467_2017_885_MOESM1_ESM.pdf]

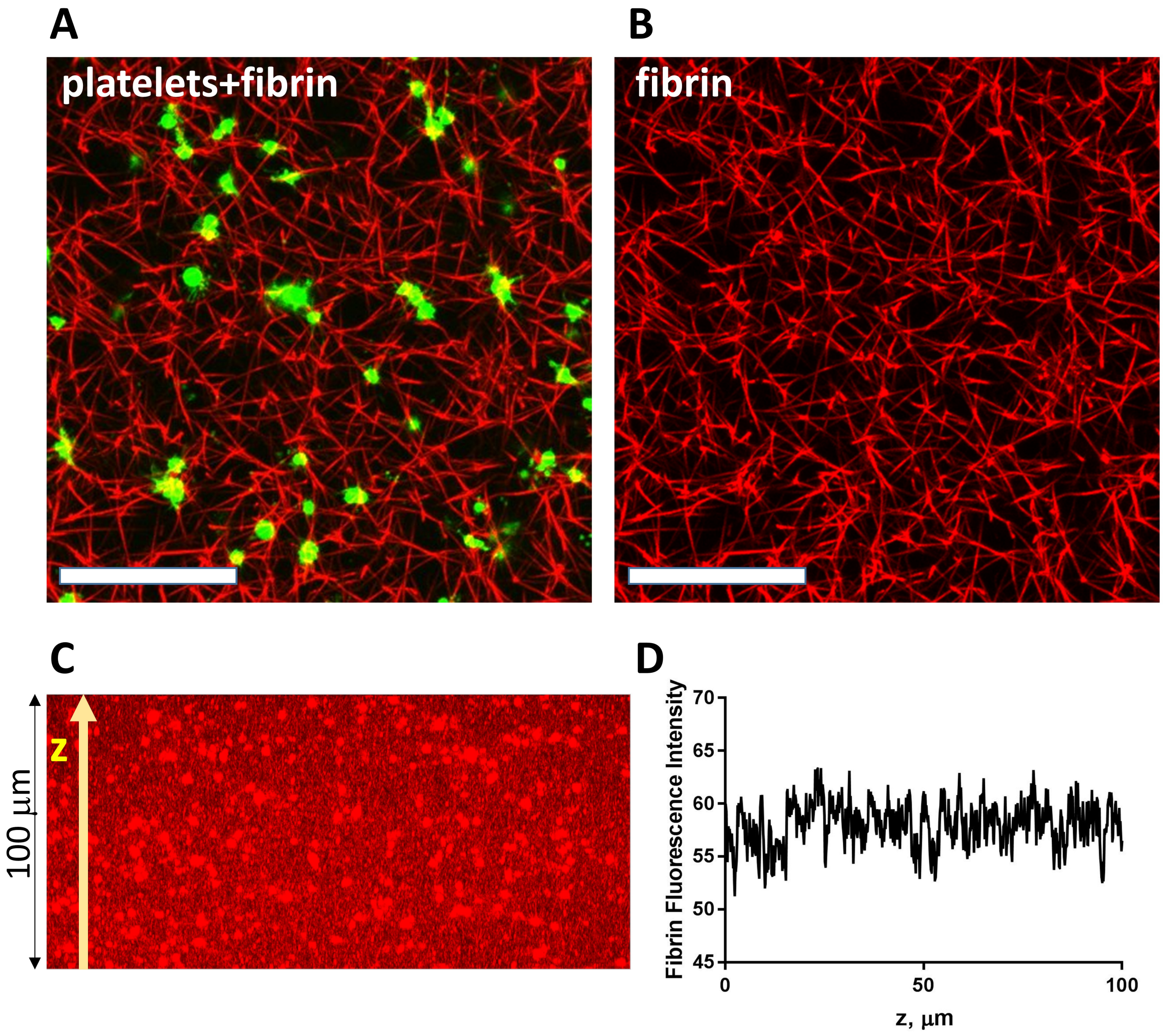

Supplementary Figure 1. A, B: Representative confocal images of a platelet-fibrin meshwork in a platelet-rich plasma clot formed and allowed to contract in the presence of abciximab (100 µg/ml). Scale bar is 30 µm. **C**: A xy-projection of fibrin in a fully contracted clot, **D**: fibrin fluorescence intensity corresponding to **C** showing no significant differences in the density of the fibrin network throughout the clot volume.

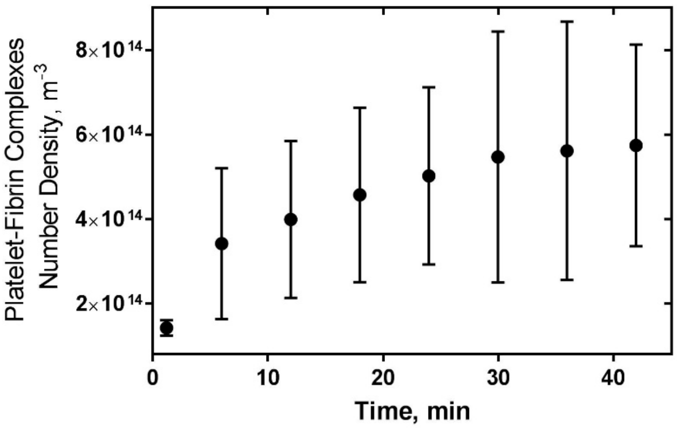

Supplementary Figure 2. Changes in concentration of platelet-fibrin complexes in the course of clot contraction (M±SD, in 3 clots).

Supplementary Table 1. Kinetic parameters and quantitative characteristics of clot contraction

I. Kinetic parameters of clot contraction

	Structure-based kinetics		*Mechanics-based kinetics*		*Displacement field-based kinetics*	
Kinetic phases	Duration, min	Rate, 1/s	Duration, min	Loading rate, Pa/min	Volumetric strain, %	Platelet moving rate, µm/s
P_1	5.0±1.2	1.07±0.05	3.9±0.1	5.6±1.3	2.4±1	0.0130±0.0012
P_2^A	28.0±1.2	0.010±0.005	11.8±0.1	4.3±0.3	9.8±4.3	0.0100±0.0009
P_2^B			31.5±0.1	3.0±1.2		
P_3	>30	0.004±0.002	>32	0.6±0.3	26.6±2.8	0.0070±0.0004

II. Quantitative characteristics of clot contraction

Parameters	*M±SD*	*N from three clot samples*
Length of a fiber kink, µm	1.6±0.5	42
Fiber retraction rate, µm/s	0.003±0.002	30
Length of a platelet filopodium, µm	2.7±1.1	300
Final area of platelet-colocalized fibrin, μm^2	5.4±0.5	540
Final size of platelet aggregates, μm^2	40.9±18.8	60
Rate of platelet displacement near the edge of the clot, µm/s	0.020±0.018	360
Rate of platelet displacement in the clot interior, µm/s	0.009±0.006	200

Supplementary Note 1

The observed platelet-fibrin dynamic rearrangements have substantial differences from the behavior of fibroblasts moving on and reshaping collagen in the extracellular matrix [1]. Although fibroblasts also pull on hand-over-hand collagen fibers and kink them, platelets use thin and short filopodia, while fibroblasts bind to collage via larger protrusions or lamellipodia [2]. Filopodia are exploratory mechanosensing devices, whereas lamellipodia provide power for movement. Fibroblast/collagen interactions involve extension of lamellipodia along the fiber, moving of the cell along the fiber and subsequent release of the fiber from the lamellipodia. In contrast, platelets do not release the displaced fibrin fibers, rather remaining attached to them and compacting fibers locally into fiber bundles and clusters, instead of crawling of the cells along the fibers. The only movement of platelets with respect to neighboring platelet-fibrin meshwork domains occurred because they were pulled along with fibrin fiber aggregates by other platelets. These marked differences between platelet-fibrin and fibroblast-collagen dynamic interactions can probably be attributed mainly to lack of polarity of platelets in contrast to fibroblasts, which have a front and rear end. Platelets do not have a system to establish such polarity, perhaps because of their smaller size and lack of nucleus. Similarly, the force field generated by platelets was shown to be isotropic [3], whereas fibroblasts generate an anisotropic force field, which enables them to migrate actively [1]. In addition, there are differences in the mechanical properties of the filamentous substrates in the platelet-fibrin and fibroblast-collagen systems underlying their structural dynamics. Fibroblasts move along collagen with an average speed of 0.069 μm/s displacing fibers at a rate of 0.04 μm/s. In contrast, platelet dynamics is slower, with the mean platelet movement rate of ~0.01 μm/s and fibrin fibers are displaced at a rate of 0.003 μm/s during clot contraction. Therefore, the platelet-fibrin interactions comprise distinct and understudied cell motility and matrix remodeling mechanisms.

Supplementary Note 2

To assess the platelet speed during clot contraction, platelets are assumed to be uniformly distributed over the clot volume of circular cross-section. Each individual platelet is assumed to be attached to the fibrin network and exert a force f_0 of a radially symmetric profile on the surrounding matrix. By neglecting the inertia term, a force balance for the platelet at distance r from the geometric center of the clot yields $F_p \sim F_c$, where $F_p \sim 2nf_0r$ – is the total force exerted on the platelet by surrounding platelets directed towards the clot center, and $F_c \sim \sigma\pi R^2/N_f$ is the resistance force due to matrix poro-viscoelastic response during contraction [4]. Here, πR^2 is the characteristic area of the clot, and N_f is the number of fibers in the clot. The compressive stress σ is estimated as $\sigma \sim (d\varepsilon/dt)/(1-\varepsilon)\kappa$, where κ is a coefficient accounting for the network morphology and viscosity of liquid (plasma), and ε is a compressive strain. Taking ε = 1-r/R, the platelet speed is given by $v=dr/dt = 2nf_0N_f(r/R)^2/(\pi\kappa)$, which therefore varies as $v \sim r^2$ and reaches its maximal value at the edge of the clot.

Supplementary References

1. Meshel, A. S., Wei, Q., Adelstein, R. S. & Sheetz, M. P. Basic mechanism of three-dimensional collagen fibre transport by fibroblasts. *Nat. Cell Biol.* **7**, 157-164 (2005).

2. Mohammadi, H., Janmey, P. A. & McCulloch, C. A. Lateral boundary mechanosensing by adherent cells in a collagen gel system. *Biomaterials* **35**, 1138-1149 (2014).

3. Schwarz Henriques, S., Sandmann, R., Strate, A. & Koster, S. Force field evolution during human blood platelet activation. *J. Cell. Sci.* **125**, 3914-3920 (2012).

4. Gibson, L. J., Ashby, M. F. (1999). Cellular solids: structure and properties. Cambridge university press.
