# Supplementary material for: Quantitative structural mechanobiology of platelet-driven blood clot contraction
Source: Nat Commun. 2017 Nov 2;8:1274. doi: 10.1038/s41467-017-00885-x (PMC5668372; doi:10.1038/s41467-017-00885-x)
Supplement: Supplementary file 3 — Description of Additional Supplementary Files [file 41467_2017_885_MOESM3_ESM.pdf]

Description of Additional Supplementary Files

File Name: Supplementary Movie 1
Description: Fibrin fiber deformation caused by a contracting platelet. Time-lapse confocal imaging of an individual contracting platelet (green) that causes bending, kinking, and condensation of a single fibrin fiber (red).

File Name: Supplementary Movie 2
Description: Fibrin fiber deformation caused by a contracting platelet. A variant of the process shown in the Supplementary Movie 1.

File Name: Supplementary Movie 3
Description: Multiple fiber deformations caused by a contracting platelet. Time-lapse confocal imaging of an individual contracting platelet causing rearrangement and compaction of multiple fibrin fibers attached to the cell.

File Name: Supplementary Movie 4
Description: Dynamics of an individual platelet filopodium during contraction. Timelapse high magnification confocal imaging of an individual platelet filopodium undergoing contraction.

File Name: Supplementary Movie 5
Description: Clusterization of contracting platelets due to compaction of the fibrin matrix. Serial confocal imaging showing clusterization of platelets due to approximation of fibrin fibers attached to the activated platelets and platelet aggregates during clot contraction. Platelets are green and fibrin is red.

File Name: Supplementary Movie 6
Description: Clusterization of contracting platelets due to compaction of the fibrin matrix. A variant of the process shown in the Supplementary Movie 5.

File Name: Supplementary Movie 7
Description: 3D reconstruction of the platelet-rich plasma clot over the course of contraction. Time-lapse 3D imaging of a contracting clot based on the confocal z-stacks acquired over the course of clot contraction near the edge of the clot, top view.

File Name: Supplementary Movie 8
Description: 3D reconstruction of the platelet-rich plasma clot over the course of contraction. Time-lapse 3D imaging of a contracting plasma clot based on the confocal z-stacks acquired over the course of clot contraction near the edge of the clot, side view.

File Name: Supplementary Movie 9
Description: 3D reconstruction of the platelet-rich plasma clot over the course of contraction. Time-lapse 3D imaging of a contracting plasma clot based on the confocal z-stacks acquired over the course of clot contraction near the edge of the clot, perspective view.

File Name: Supplementary Movie 10
Description: 3D reconstruction of the plasma clot over the course of contraction. Time-lapse 3D imaging of a contracting plasma clot based on the confocal z-stacks acquired over the course of clot contraction in the clot interior, perspective view.

File Name: Supplementary Movie 11
Description: 3D reconstruction of the platelet-rich plasma clot over the course of contraction. Time-lapse 3D imaging of a contracting plasma clot based on the confocal z-stacks acquired over the course of clot contraction in the clot interior, side view.
